# Non-Invasive Monitoring of Corticosterone Levels in Mouse Urine with Liquid Chromatography-Electrospray Ionization-Tandem Mass Spectrometry

**DOI:** 10.3390/molecules29225399

**Published:** 2024-11-15

**Authors:** Laura Howe-Wittek, Paul Kroschk, Kai Nieschalke, Harshadrai M. Rawel, Stephanie Krämer, Jens Raila

**Affiliations:** 1 Institute of Nutritional Science, University of Potsdam, 14558 Nuthetal, Germany; laura.wittek@hmu-potsdam.de (L.H.-W.); kai_nieschalke@yahoo.de (K.N.); harshadrai.rawel@uni-potsdam.de (H.M.R.); 2 Department of Medicine, Health and Medical University, 14471 Potsdam, Germany; 3 Federal Office of Consumer Protection and Food Safety (BVL), Unit 502-European Union Reference Laboratory (EURL) for Veterinary Drug Residues, 12277 Berlin, Germany; 4 Interdisciplinary Center of 3Rs in Animal Research (ICAR3R), Clinic of Veterinary Medicine, Justus Liebig University of Giessen, 35392 Giessen, Germany; stephanie.kraemer@vetmed.uni-giessen.de

**Keywords:** corticosterone, urine, liquid chromatography-electrospray ionization-tandem mass spectrometry, enzymatic hydrolysis, liquid–liquid extraction, C57BL/6J mice, metabolic cage, stress, non-invasive monitoring

## Abstract

An innovative method for the quantification of corticosterone in the urine of C57BL/6J mice by liquid chromatography-electrospray ionization-tandem mass spectrometry was developed. Unconjugated and glucuronidated corticosterone was detected in the urine samples using enzymatic hydrolysis following liquid–liquid extraction. After optimization of the extraction protocol and LC-MS/MS parameters, we performed a validation study using a representative urine pool of C57BL/6J and Naval Medical Research Institute mice. The method shows good linearity (1–5000 fmol/µL) and the calculated limit of quantification amounts to 0.823 fmol/µL. Both intra-day and inter-day variation was ≤10%, while their recoveries amounted to 90.4–110.6% and 99.8%, respectively. Twenty-four hour urine collection of C57BL/6J mice restrained in two different metabolic cage types for two times was used to test the validated method. To control the hydration level of mice, the corticosterone concentration in their urine was normalized to urinary creatinine concentration. Our LC-MS/MS method represents a highly specific analytical tool for the quantification of corticosterone levels in urine samples, assisting in non-invasive monitoring of acute stress levels in laboratory mice.

## 1. Introduction

The hypothalamic-pituitary-adrenocortical (HPA) axis is a complex regulatory circuit of the endocrine organ hypothalamus, pituitary gland, and adrenal cortex. The glucocorticoid corticosterone (11,21-dihydroxy-4-pregnene-3,20-dione) is a functional parameter of the *Zona fasciculata* cells located in the adrenal cortex, and it represents the major stress hormone of mice. Corticosterone depicts the precursor of the mineralocorticoid aldosterone and activates glucocorticoid as well as mineralocorticoid receptors. Stress monitoring of experimental animals is becoming more relevant in laboratory animal science to enhance their well-being within the bounds of possibility. In addition to the ethical responsibility for the laboratory animals, an increased activity of the HPA axis results in elevated glucocorticoid levels in the systemic circulation. An increase in glucocorticoids has an impact on multiple physiological functions, for example, the fluid and electrolyte balance as well as the cardiovascular system (vasoconstriction); fat (lipolysis), protein (proteolysis), and muscle metabolism (inhibition of muscle growth); and behavior [1,2,3,4,5].

In laboratory animal science, there is a growing concern that scientific findings cannot be consistently replicated by independent studies. This issue is designated as ‘reproducibility crisis’ and addresses various methodological weaknesses in experiments with animals [6,7]. It is essential to mention that not only the applied experimental method, but also the housing conditions of mice have a significant impact on the study outcome. Enrichment of the cage environment contributes to the normalization of the data, considering the mouse strain, age, and sex, among other housing attributes [8]. The 3R principle was first introduced in 1959 with the aim to improve animal welfare and to reduce the utilization of animals in scientific research. This principle is split into the following three main components: replacement, reduction, and refinement [9]. The refinement aspect of the 3Rs primarily contributes to the stress reduction in mice while focusing on the improvement of animal well-being. Examples for refinement strategies include “enriched housing environments that reduce stress and encourage natural behaviors, non-restraint methods in handling and training, refined dosing and sampling techniques that prioritize animal comfort, the critical role of optimal pain management, and the importance of regular animal welfare assessment in maintaining the rodents’ well-being” [10]. In any case, a multidimensional approach should be taken to assess the animals’ condition during an experimental intervention including physiological, behavioral, and biochemical parameters. The quantification of corticosterone in urine, feces or hair represents a biochemical parameter, which is observer independent, non-invasive, and easy to perform, in accordance with a thorough validation process [11,12,13].

The liver metabolizes most of the free glucocorticoids, in contrast to glucocorticoids that are bound to corticosteroid-binding globulin or to albumin. Corticosterone is poorly water-soluble and can be transferred into a more water-soluble metabolite through biotransformation processes such as oxidation at position 11β, reduction at position 20, A-ring reduction, and conjugation reactions [14]. Sulfonation and glucuronidation represent the main types of conjugation reactions in the context of steroid biotransformation. Steroid conjugates are excreted in the urine and bile, but excretion of unconjugated steroids in the urine is low. In the context of urinary excretion, the kidney cells take up the steroid conjugates circulating in the blood via two transport mechanisms: organic anion symport or exchange. An electrochemical gradient is thereby used to reach the lumen of the nephron [15,16,17]. Steroids bound to proteins are not present in the urine under physiological conditions, because these are priorly excluded by kidney filtration. Therefore, urinary corticosteroid concentrations represent the free hormone fraction in blood over time [18]. As corticosterone secretion follows a daily rhythm, urine should be sampled for at least 24 h. To also circumvent bacterial decay, frozen urine is recommended to be stored [4,16,17,18]. Urine from laboratory mice can be gathered using the following methods: cages possessing a grid floor such as metabolic cages, mice can be placed on clear plastic wrap or held over a petri dish. Modifications to these methods have also been reported [19,20,21]. Additionally, hydrophobic sand can be utilized as an alternative bedding material in contrast to the metabolic cage restraint for urine sampling of laboratory rodents [21,22].

In the frame of stress monitoring during experiments, corticosterone levels in urine samples of laboratory rodents are frequently measured. Commercial kits or self-developed assays are commonly used for quantification of glucocorticoids in urine, which are based on competitive immunoassays, radio immunoassays (RIA), and enzyme immunoassays (EIA) [4,17,23]. Not the assay sensitivity, but the assay specificity of the immunoassays poses a challenge, as structurally related compounds in urine can cause cross reactions [5,18]. High-performance liquid chromatography tandem mass spectrometry (HPLC-MS/MS) depicts a highly specific but also sensitive analytical method, as compounds with very low contents in biological matrices can be analyzed, a small sample volume is required, and multiple analytes can be simultaneously determined in a single analytical run. In comparison with immunoassays, the HPLC-MS/MS methodology provides a larger linear dynamic range and higher accuracy, because internal standards are used, omitting the application of immunological reagents [5,24,25].

In fact, there is currently no published and validated LC-MS/MS method for the quantification of corticosterone in mouse urine. We are acquainted with bioanalytical methods for the quantification of corticosterone besides other endogenous compounds in serum, plasma, and hair of mice. These methods include, among others, LC-MS/MS [4,26], LC-atmospheric pressure chemical ionization-(APCI)-MS/MS [13], and LC coupled with high-resolution accurate mass spectrometry (HRMS), using a quadrupole time-of-flight (Q-TOF) system [27]. The aim in the present study was to establish and validate a non-invasive LC-MS/MS method for corticosterone quantification to implement the refinement aspect of the 3R guiding principle [28]. We, therefore, chose urine as sample matrix for analyses, because blood sampling and shaving off fur cannot be considered non-invasive sampling methods [4,18]. Twenty-four-hour urine collection of mice restrained in metabolic cages was utilized for method establishment and validation. Elevated corticosterone levels in urine samples of mice can be assumed, which, in turn, can be attributed to an increased stress potential due to the barren environment and mainly reflecting the functional construction of the metabolic cages [12,29,30,31]. We believe that the development of new methodological protocols for quantification of corticosterone levels in urine samples of mice will allow us to better understand the condition of the individual animal to reduce its stress level for the purpose of generating more reproducible and justifiable data.

## 2. Results

**Scheme 1:**
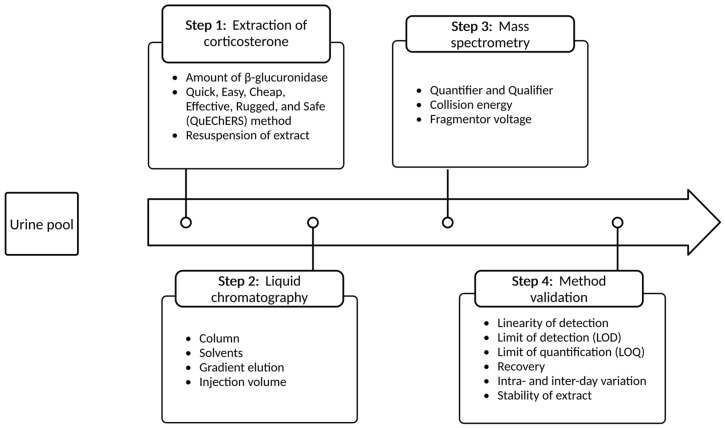
Workflow of method development; including liquid–liquid extraction (step 1), liquid chromatography (step 2), mass spectrometry (step 3), and method validation (step 4). The same urine pool was used for all testing.

The developed protocol for corticosterone quantification in mouse urine was comprehensively validated (see Scheme 1). This method was adapted from Hauser, Deschner, and Boesch [32].

### 2.1. Extraction of Corticosterone from Mouse Urine

#### 2.1.1. Amount of β-Glucuronidase

Enzymatic hydrolysis with β-glucuronidase from *E. coli* was conducted to cleave the corticosterone glucuronide in urine. To check whether sufficient enzyme quantity is provided for deconjugation, the yield of unconjugated corticosterone was determined. Therefore, the following quantities of β-glucuronidase were tested: 25 U, 50 U, 100 U, 200 U, and 400 U.

Different amounts of enzyme were added to a 200 µL aliquot of the urine pool and the extraction was conducted as described in Section 4.4. Each enzyme concentration was tested in quadruplicate. For unconjugated corticosterone, the percentage variation of all tested amounts of β-glucuronidase was ≤8.76% in reference to the urine pool, which was subjected to 200 U enzyme (see Table 1). Thereafter, the results indicated that the addition of 200 U of β-glucuronidase to urine samples was sufficient to completely cleave different concentrations of glucuronidated corticosterone, which might be present within the biological range of variation.

#### 2.1.2. Quick, Easy, Cheap, Effective, Rugged, and Safe (QuEChERS) Method

A liquid–liquid extraction was performed consisting of an aqueous phase (sodium phosphate buffer, mouse urine, β-glucuronidase prepared in HPLC suitable water and an organic phase (methyl tert-butyl ether). To check whether the Quick, Easy, Cheap, Effective, Rugged, and Safe (QuEChERS) method could further improve the extraction protocol, magnesium sulfate (MgSO_4_) and sodium chloride (NaCl) were added to samples after the enzymatic hydrolysis with β-glucuronidase. The QuEChERS method was initially established for the extraction of pesticide residues from fruits and vegetables by Anastassiades and Lehotay [33] and was later combined with both gas chromatography and liquid chromatography as well as tandem mass spectrometric detection (GC-MS/MS, LC-MS/MS) by Payá et al. [34]. A clearer phase separation with the help of the QuEChERS method could be achieved by salting out an organic substance from an aqueous solution. This is based on the increase in polarity of the aqueous phase, as the salts dissolve better in water than in the organic substance. The aqueous phase is thereby saturated. At the same time, the change in polarity allows less polar analytes to be completely dissolved in the organic phase and displaced from the aqueous phase. In the partitioning step, we tested the following amounts of MgSO_4_ and NaCl: 640 mg MgSO_4_ and 160 mg NaCl, 440 mg MgSO_4_ and 105 mg NaCl, 240 mg MgSO_4_ and 60 mg NaCl.

The ratios of the salt concentrations to the quantity of sample in the publication of Anastassiades and Lehotay [33] were adapted to the sample volumes used in this work. The urine pool samples were spiked with external corticosterone standard prior extraction modifications with or without salt (*m*/*z* 347.2 > 329.3, 347.2 > 311.2, 347.2 > 293.2, 347.2 > 135.0, 347.2 > 121.1, 347.2 > 97.1). When the recovery of external corticosterone standard in extracted samples with salt addition was directly compared with no salt addition, the recovery of corticosterone without salt addition was found to be clearly higher.

The lowest salt quantity (240 mg MgSO_4_∙7H_2_O and 60 mg NaCl) achieved the highest recovery of 94.3%, as opposed to the higher salt quantities. The recovery of corticosterone in extracted samples including no salt addition amounted to 98.2%. In the final protocol, the aqueous phase was separated from the organic phase (ether phase) by freezing it out of solution (−21 °C, 30 min).

#### 2.1.3. Resuspension of Extract

The ratio of acetonitrile to water and the volume for resuspension of evaporated extracts were also checked. We tested 30% acetonitrile and 80% acetonitrile, both acidified with 0.1% formic acid, and a volume of 100 µL versus 500 µL. A volume of 1000 µL of 80% acetonitrile/0.1% formic acid was additionally checked. The highest recovery was obtained with 500 µL of 80% acetonitrile/0.1% formic acid. In detail, recovery of samples resuspended in 100 µL of 80% acetonitrile/0.1% formic acid amounted to 135% while recovery of samples resuspended in 30% acetonitrile/0.1% formic acid amounted to 104% with regard to the respective one-point calibration. 

### 2.2. Development of a LC-MS/MS Method for Quantification of Corticosterone in Mouse Urine

#### 2.2.1. Liquid Chromatography

The chromatographic conditions were optimized while considering baseline separation, peak height, maximum sensitivity, signal intensity, and signal to noise ratio (SNR) of the MS detection. First, a Kinetex C18 column (2,6 μm, 100 Å, 100 × 30 mm; Phenomenex Ltd., Aschaffenburg, Germany) was compared with a Kinetex C8 column (2.6 µm, 100 Å, 150 × 4.60 mm; Phenomenex Ltd., Aschaffenburg, Germany). Considering the aforementioned parameters, the Kinetex C8 column with a flow rate of 0.450 mL min^−1^ provided the best separation (see Table 2). The solvent composition and gradient elution suggested by Hauser, Deschner, and Boesch [32] were modified. Eluent A was composed of water and 0.1% formic acid instead of water/acetonitrile (95/5, *v*/*v*). Eluent B contained acetonitrile and 0.1% formic acid, as opposed to acetonitrile/water (95/5, *v*/*v*). Eluent A and B in the publication of Hauser, Deschner, and Boesch [32] were also acidified with 0.1% formic acid. The gradient elution of our established method was performed at a flow of 0.450 mL min^−1^: 20% B (0–1 min), linear increase to 100% B (1–8 min), maintained at 100% B (8.01–9 min), followed by a linear decrease to 20% B (9.01–12 min) and a postrun at 20% B (12.01–16 min). Accordingly, the final gradient differed significantly from the one reported by Hauser, Deschner, and Boesch [32]. The injection volume of 20 µL [32] was decreased to 5 µL after testing 2.5 µL, 5 µL, and 10 µL considering the smaller amount of mouse urine available compared to primate urine. The signal intensity and the separation of the peaks in the chromatogram were sufficient at 5 µL level.

#### 2.2.2. Mass Spectrometry

ESI(+)-MS/MS in MRM mode was applied for quantification of corticosterone in mouse urine samples. Collision-induced dissociation studies were conducted at first to determine the fragmentation of the protonated molecular ions. In this way, the required collision energies for fragmentation into respective product ions were determined to achieve optimum intensity for each MRM transition. The fragmentor voltage for specific precursors was also optimized to achieve maximum sensitivity. Solely one mass transition (quantifier) was applied for quantification (corticosterone, *m*/*z* 347.2 > 97.1; corticosterone-d8 *m*/*z* 355.3 > 100.2). An additional mass transition, the qualifier, was included in analyses for unequivocal identification (corticosterone, *m*/*z* 347.2 > 121.1; corticosterone-d8, *m*/*z* 355.3 > 125.0).

**Scheme 2:**
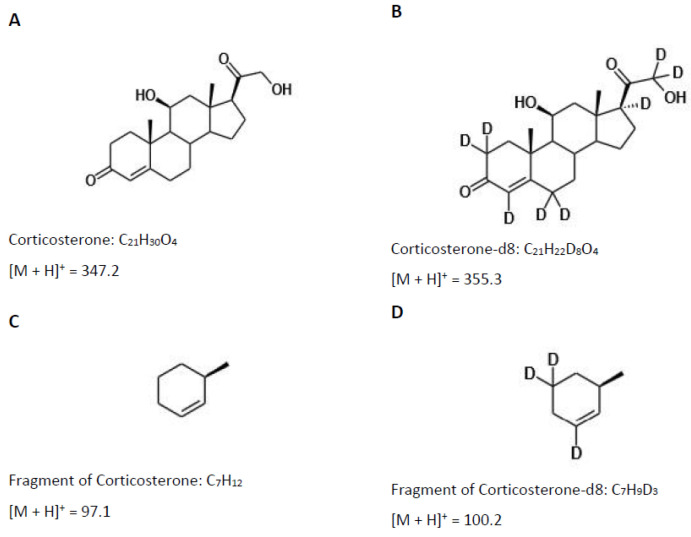
Chemical structures of corticosterone (**A**) and corticosterone-d8 (**B**) as well as their suspected product ions (**C**,**D**) used for MRM quantification.

The peak area of the analyte was set into relation with the peak area of the deuterated internal standard (relative quantification). Scheme 2 illustrates the chemical structure of corticosterone and the deuterated internal standard corticosterone-d8, including its fragmentation reaction yielding the most abundant product ions of corticosterone.

### 2.3. Analytical Procedure Verification 

#### 2.3.1. Results of Linearity of Detection, LOD, LOQ

The response for corticosterone in urine samples was linear up to 5000 fmol/µL (y = 7.3266x + 1831.7, r^2^ = 0.988, see Table 3). The mean concentration of corticosterone in female and male urine samples of C57BL/6J mice amounted to 20.68 fmol/µL while the minimum and maximum concentration was 4.04 fmol/µL and 47.67 fmol/µL, respectively. The limit of detection (LOD) for corticosterone in mouse urine was calculated to 0.358 fmol/µL and the limit of quantification (LOQ) amounted to 0.823 fmol/µL (y = 3.0101x − 2.3814, r^2^ = 0.987).

#### 2.3.2. Results of Recovery, Intra- and Inter-Day Variation

Recovery, intra- and inter-day variation calculations were based on samples from a urine pool spiked with the same amount of corticosterone-d8 (10 µL of 1 pmol/µL reagent) before extraction. Both intra-day (n = 3) and inter-day (n = 9) variation was ≤10% and the variation acceptance criterion was therefore met (see Section 4.8.2). Recoveries of intra- and inter-day variation amounted to 90.4–110.6% and 99.8%, respectively.

#### 2.3.3. Results of Accuracy and Matrix Effect

The percentage accuracy of the applied protocol was calculated based on solvent spiked with the internal standard corticosterone-d8 at five different concentrations. The actual concentration of each point of the calibration curve was then calculated using the linear equation. The percentage accuracy for the method described here was 97.9%.

The extent of matrix effects on ionization was determined by comparing the response of the internal standard spiked at five different concentrations into either urine pool samples prior extraction or solvent. The calculated matrix effect amounted to 15.1% based on the slopes of the linear regression analyses.

#### 2.3.4. Results of Stability of Extracts

For a long-term storage time between 28 d and 34 d, the area under the curve of extracted corticosterone in 80% acetonitrile/0.1% formic acid decreased by 13.9–29.7% (*m*/*z* 347.2 > 97.1). Therefore, aliquots of the extracted urine samples were directly analyzed via the established and validated LC-MS/MS method.

### 2.4. Corticosterone and Creatinine Concentrations in Urine of Stressed Mice

**Figure 1:**
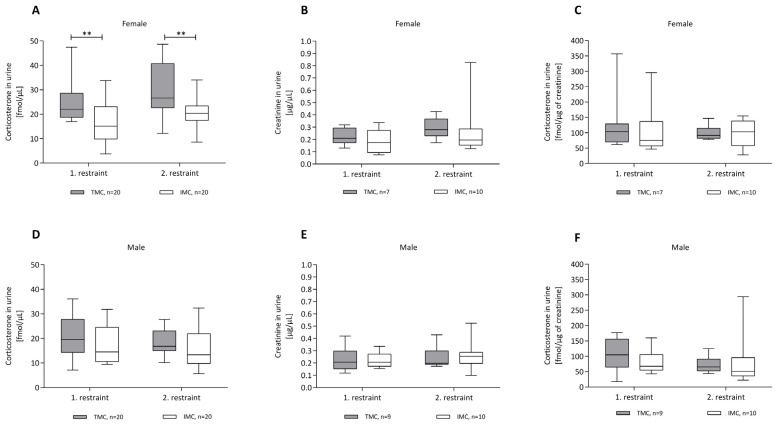
Corticosterone concentration in urine [fmol/µL] (**A**,**D**), creatinine concentration in urine [µg/µL] (**B**,**E**), and corticosterone concentration in urine [fmol/µg of creatinine] (**C**,**F**) during first and second restraint in either Tecniplast metabolic cage (TMC) or Innovative metabolic cage (IMC). Differences between TMC vs. IMC were calculated by independent-samples *t* test or Mann–Whitney U test and analyzed separately for each point in time. ** *p* ≤ 0.01. The data are presented as mean (standard deviation).

The present LC-MS/MS method for the analysis of corticosterone levels in mouse urine is based on the publication of Hauser, Deschner, and Boesch [32], which was initially established for quantification of steroids in primate urine. As described in Section 4.4, we extracted the glucuronidated fraction of corticosterone metabolites besides unconjugated corticosterone. For female mice, significantly higher corticosterone concentrations in urine during the 24 h restraint in the Tecniplast metabolic cage (TMC) relative to the 24 h restraint in the Innovative metabolic cage (IMC) were detected (*p* ≤ 0.01, see Figure 1A). No effects of the tested metabolic cage types on the urinary corticosterone secretion of male mice could be detected (see Figure 1D). The creatinine concentration in urine was also determined to control the effects of dilution. The corticosterone concentration in urine can vary among individuals which may be associated with the hydration level and not solely with their stress level.

No significant differences in creatinine concentrations were detected for both sexes when comparing cage types after both restraints (see Figure 1B,E). The ratio of urinary corticosterone to urinary creatinine also showed no differences between the TMC and the IMC. This was true for both sexes and restraints (see Figure 1C,F).

### 2.5. Water Intake and Urinary Excretion of Stressed Mice

**Figure 2:**
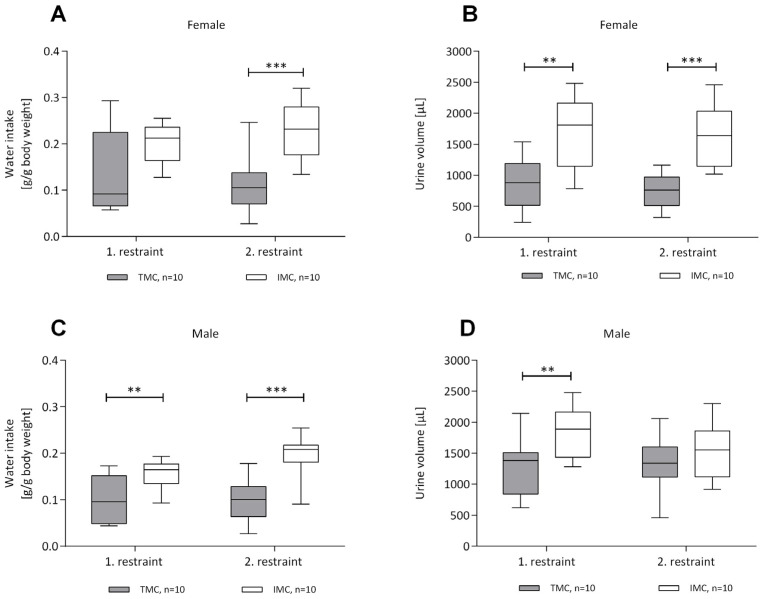
Water intake [g/g body weight] (**A**,**C**) and excreted urine volume [µL] (**B**,**D**) during first and second restraint in either Tecniplast metabolic cage (TMC) or Innovative metabolic cage (IMC). Differences between TMC vs. IMC were calculated by independent-samples t test or Mann–Whitney U test and analyzed separately for each point in time. ** *p* ≤ 0.01, *** *p* ≤ 0.001. The data are presented as mean (standard deviation).

During the second restraint, both sexes consumed significantly more water during IMC restraint relative to TMC restraint (*p* ≤ 0.001, see Figure 2A,C). Water intake was also significantly higher for male mice during first restraint in the IMC compared with the TMC (*p* ≤ 0.01, see Figure 2C). The higher water consumption during the first (only for males) and second restraints in the IMC (both sexes) was associated with a higher urine output during the first (both sexes) and second restraints (only for females, see Figure 2B,D). At both points in time, females excreted significantly more urine in the IMC compared to restraint in the TMC (1st restraint: *p* ≤ 0.01, 2nd restraint: *p* ≤ 0.001; see Figure 2B). However, this could only be confirmed for the males during the first restraint (*p* ≤ 0.01, see Figure 2D). The observed cage-dependent differences in water intake confirm the relevance of normalizing urinary corticosterone concentration to creatinine.

## 3. Discussion

The data in the study at hand are not directly transferrable to other studies, because we are publishing this LC-MS/MS method for quantification of corticosterone concentrations in mouse urine for the first time. It should be emphasized that this innovative LC-MS/MS method is highly specific in contrast to conventional enzyme and radio immunoassays, which is attributable to the fact that LC/MS-MS technology detects molecules possessing a specific mass-to-charge-ratio [4,17,27]. Importantly, our method ensures a non-invasive monitoring of stress levels in mouse urine during experimental procedures.

After 1 h restraint stress in a 50 mL conical tube, Chu et al. [13] analyzed corticosterone concentrations in plasma samples of male and female BALB/c mice via LC-APCI-MS/MS. They measured mean plasma corticosterone concentrations of 541 fmol/μL for stressed males [13]. In contrast, the maximum corticosterone concentration in urine determined in the present study amounted to 47.67 fmol/µL. Therefore, the corticosterone concentrations published by Chu et al. [13] were approximately a hundred times higher than our measured corticosterone concentrations. The comparability of the different sample matrices (plasma vs. urine), measuring tools (LC-APCI-MS/MS vs. LC-ESI-MS/MS) as well as the study design (1 h vs. 24 h restraint stress) need also to be taken into account. BALB/c mice were acutely stressed (conical tube) while C57BL/6J mice in this study were rather chronically stressed (metabolic cages). The LODs and LOQs of the present study (LOD = 0.358 fmol/µL, LOQ = 0.823 fmol/µL), compared with the study of Chu et al. [13] (LOD = 0.578 fmol/µL, LOQ = 1.730 fmol/µL), are approximately in the same range.

In addition to the enzymatic hydrolysis with β-glucuronidase, a solvolysis of urine samples could also be incorporated into the extraction protocol. In detail, the frozen aqueous phase after phase separation could be thawed and subjected to solvolysis. In this work, the presented results stem from analyses of the ether phase, which was decanted. A solvolysis protocol of mouse urine samples based on the publication of Hauser, Deschner, and Boesch [32] was actually tested, but we did not validate this method so far. The amount of corticosterone detected after solvolysis was lower than after enzymatic hydrolysis, which mainly represents the glucuronidated corticosterone metabolites. Interestingly, we found a higher sulfuronidated corticosterone content for female C57BL/6J mice than for males. For females, corticosterone sulfate on average amounted to 13.71 fmol/µL and 16.29 fmol/µL after first restraint in TMC and IMC respectively. In contrast, for males, corticosterone sulfate on average amounted to 5.34 fmol/µL and 3.64 fmol/µL after first restraint in TMC and IMC respectively. Steroid sulfates, such as corticosterone 21-sulfate, have already been detected in mouse urine and take part in chemical communication and physiological signaling [35]. An underestimation of the quantified corticosterone concentrations in the urine of mice applying the validated protocol can therefore not be entirely eliminated. After experimental peroxisome proliferator-activated receptor activation and acetaminophen intoxication in a study with mice, two corticosterone metabolites, 11β-hydroxy-3,20-dioxopregn-4-en-21-oic acid (HDOPA) and 11β,20α-dihydroxy-3-oxopregn-4-en-21-oic acid (DHOPA), were detected in urine samples via UPLC-TOFMS. HDOPA and DHOPA represent oxidized corticosterone metabolites and could be applied as urinary stress biomarkers including the stimulation of the adrenal cortex through the HPA axis, i.e., the stress axis. Most of the biotransformation processes take place in liver tissue. In this context, it is further suggested that the liver is decisive for the metabolism of corticosterone to 21-carboxylic acids in mice [36]. In detail, HDOPA is formed from corticosterone after multiple reaction steps while DHOPA represents the end product of corticosterone biotransformation. These 21-carboxylic acids, originating from liver tissue, are then excreted into the urine via the kidneys [36,37,38]. In a study of Beyoğlu et al. [38], further corticosterone metabolites; 5α-dihydrocorticosterone and 3α,11β,20α-trihydroxypregn-4-en-21-oic acid; were detected in mouse urine after acetaminophen intoxication (UPLC-ESI-QTOFMS). We identified 5α-dihydrocorticosterone, HDOPA, DHOPA, and 3α,11β,20α-trihydroxypregn-4-en-21-oic acid as endogenous biomarkers of acetaminophen hepatotoxicity in mouse urine and the affected pathway was assigned to the ‘stress response’ [38]. In the context of a study on Huntington’s Disease, hydroxycorticosterone and hydroxy-dehydrocorticosterone were identified as corticosterone metabolites in mouse urine (UHPLC-HRMS, ESI+) [39]. The main challenge is to determine which corticosterone metabolites are solely xenobiotic-related and which of them are endogenous biomarkers to select suitable stress markers. The basic question is therefore which approach is most accurate for the detection of corticosterone and its metabolites in the urine of mice using mass spectrometry? We suggest that enzymatic hydrolysis and solvolysis in the extraction protocol (1) and the detection of the *m*/*z* of corticosterone and known corticosterone metabolites in mass spectrometry analysis (2) might be combined in a single run. With this approach, the detection of corticosterone and its metabolites in urine samples of mice is probably highest. In the last step of our extraction protocol, the ether phase was fully evaporated, and the residue was re-suspended afterwards. The evaporation step could be shortened to avoid loss of analytes due to overdrying the samples [5]. In future experiments, the recovery of the analyte could be checked by drying samples to a defined volume. In the present study, we tested the resuspension of the extract including the ratio of acetonitrile to water and the volume for resuspension of evaporated extracts.

The glucuronidated fraction of corticosterone as well as unconjugated corticosterone was quantified in the 24 h urine collection. Concerning both housing periods, female mice showed significantly elevated urinary corticosterone concentrations during TMC as opposed to the IMC restraint. For males, no significant differences between both metabolic cage types could be detected for neither of the two points in time. Interestingly, it was shown before that the adrenal weight varies depending on the gender of rodents. In a study with F1 offspring of Naval Medical Research Institute mice crossed with C57BL/6J mice, greater cortex volumes, a higher adrenal gland weight, and a larger *Zona fasciculata* was assigned to the female mice as opposed to the male mice [40].

Whether the increased corticosterone levels in female urine samples are attributable to sex differences, or indicate a higher stress level of females, leads to the discussion of a sex-dependent excretion route of corticosterone in mice. Touma et al. [17] demonstrated that the route of steroid excretion is indeed different between male and female mice. Radio-labelled corticosterone was intraperitoneally injected into female and male C57BL/6J mice. Urine and fecal samples of both sexes were collected afterwards. Regarding the urinary excretion, females excreted 43.8 ± 4.4% of metabolized corticosterone while males only excreted about 28.3 ± 4.0% [17]. In another study of Kalliokoski et al. [41], BALB/c mice were intravenously injected with corticosterone while female mice were shown to excrete 60% of its metabolites in urine and 40% in feces. It should also be mentioned that the urinary creatinine concentration and urinary corticosterone as a ratio to urinary creatinine depicted no differences between both tested metabolic cage types and this tendency was shown for both sexes after both restraints. Creatinine is a breakdown product of the skeletal muscle peptide creatine phosphate produced at a constant rate. It is, therefore, often used for correction of dilution effects of urinary biomarkers, because creatinine levels are not modified by urine volume [18]. Metabolic cages possess a primarily functional construction for the purpose of urine and feces collection as well as the monitoring of food and water consumption [29,30,42]. We thus gathered data including urine volume and water intake in both metabolic cage types. A cage-dependent water intake was observed while water intake in the IMC tended to be or was significantly higher compared with the TMC (see Section 2.5). Accordingly, a dilution effect of the urinary corticosterone levels in the IMC could be assumed. On the other hand, the mice tended to consume less water in the TMC, which could have led to a higher concentration of corticosterone in urine. This explanation could be applicable to the data of female mice because the total urinary corticosterone levels, but not the normalized urinary corticosterone concentrations, were significantly higher in the TMC compared with the IMC. The male mice, in particular, showed no differences between the metabolic cage types regardless of whether the total corticosterone levels or the corticosterone concentrations normalized to creatinine were considered.

We additionally measured corticosterone metabolites in dried fecal samples (FCM) of the same study in our previous publication via an enzyme immunoassay [12]. Interestingly, no significant differences in FCM levels were detected between both metabolic cage types. This was true for both restraints and sexes. It can therefore be concluded that the established LC-MS/MS method in this publication not only provides a more specific methodology than the previously applied enzyme immunoassay, but is also consistent with the data gathered beforehand. Taken together, (1) an intrinsically elevated stress level of female mice, (2) a sex-dependent excretion route of corticosterone, and (3) the hydration level of mice might represent aspects to ensure a detailed and differentiated analysis of our urinary corticosterone data. Even if the collection and drying of fecal pellets is more convenient, it should be noted that feces can be cross-contaminated with urine and that mice engage in coprophagy. Also, hepatic metabolites ending up in feces are prone to microbial degradation.

It can therefore be assumed that urine analysis is more straight forward although quantitatively less steroids are excreted from mice via urine [14,17]. In the frame of stress monitoring during experiments, acute stress levels of the animal should be assessed to focus on a defined time-point or short-term patterns of steroid secretion [5]. For mice, the lag time for corticosterone secretion in urine amounts to approximately 2 h after intraperitoneal injection of radio-labelled corticosterone while for feces a lag time between 4 h and 10 h (depending on the activity level) was described [17]. Consequently, not only the effect of intestinal microorganisms on corticosterone levels, but also the intestinal passage time must be taken into account for stress monitoring in fecal samples. In addition, the sample preparation of feces for LC-MS/MS detection requires more extensive purification steps and is therefore time-consuming due to the lipophilic nature and complex composition of this biological matrix. Matrix effects can further affect the accuracy of LC-MS/MS analyses including ion suppression or enhancement. Fecal samples have indeed been described for their significant matrix effects due to interfering substances that are particularly challenging to remove during sample preparation [5,14,43,44]. However, corticosterone analysis in urine samples can also entail drawbacks. First, mice need to be housed individually and special cage systems, such as metabolic cages, need to be utilized for urine collection [4]. Second, urine cannot be collected from control animals without additional handling or acclimatization to hydrophobic sand [19,22]. Third, the analysis of corticosterone and its metabolites in urine samples can solely be applied for acute stressors. Overall, LC-MS/MS represents a state-of-the-art technology that can be safely adopted for corticosterone analysis, provided that the method is specifically established and validated for the respective species and sample matrix.

## 4. Materials and Methods

Unconjugated and glucuronidated corticosterone was extracted from 40 mouse urine samples collected during two 24 h restraints in each metabolic cage type and was subsequently quantified via LC-MS/MS.

### 4.1. Materials and Standards

Water suitable for HPLC (HiPerSolv CHROMANORM, VRW International GmbH, Darmstadt, Germany), sodium dihydrogen phosphate and disodium phosphate (anhydrous, Altman Analytik GmbH & Co. KG, Munich, Germany), sodium carbonate (anhydrous, Carl Roth GmbH & Co.KG, Karlsruhe, Germany), methyl tert-butyl ether (HiPerSolv CHROMANORM, VWR International GmbH, Darmstadt, Germany), acetonitrile (LiChrosolv, hypergrade for LC-MS, Sigma-Aldrich Chemie GmbH, Taufkirchen, Germany), and formic acid (≥99%, for LC-MS, VWR International GmbH, Darmstadt, Germany) were utilized for extraction and subsequent LC-MS/MS analysis. Corticosterone standard (≥98% purity; Cayman Chemical, Ann Arbor, MI, USA) and corticosterone-d8 standard (98% purity; Toronto Research Chemicals, Toronto, Canada) were prepared in 100% acetonitrile in different concentrations and stored at −20 °C.

### 4.2. Stress Protocol

To investigate the HPA response to stress stimuli, the levels of unconjugated and glucuronidated corticosterone in mouse urine were quantified after 24 h restraint and social stress, i.e., metabolic cage housing. Female (n = 20) and male (n = 20) mice were single-housed in two different metabolic cage types (TMC: Tecniplast metabolic cage, IMC: Innovative metabolic cage) for two times in total. After the first 24 h restraint, females were returned to their home cages in groups of five animals while males were single-housed until the end of the experiments. A 6 d resting period was integrated followed by a second restraint according to the same experimental workflow.

### 4.3. Urine Samples and Water Intake

24-h urine collected from C57BL/6J mice was transferred into 2 mL polypropylene tubes with a pipette to determine the total volume. Urine samples were stored at −80 °C until analysis. A detailed description of animals and housing conditions, metabolic cages, and the study design can be found in a previous publication (Wittek et al. [12]). The urine pool used for method validation stemmed from female and male 66 d to 364 d old C57BL/6J and NMRI mice housed in both metabolic cage types for two and four times in total. Water intake was manually measured over a period of 24 h during restraint in both metabolic cage types. The empty weight of each water bottle was included in calculations.

### 4.4. Extraction Protocol for Corticosterone from Mouse Urine

To extract corticosterone, 200 μL aliquots from 24-h urine collections were used. β-Glucuronidase type VII-A from *E. coli* (25,000 U, Sigma-Aldrich Chemie GmbH, Taufkirchen, Germany) was utilized for enzymatic hydrolysis of glucuronidated corticosterone. Prior analysis, the lyophilized enzyme was dissolved in 5 mL of HPLC-grade water. A total of 800 μL of 0.25 M sodium phosphate buffer (0.25 M, pH 6.9), 40 μL of β-glucuronidase (200 U), and 10 μL of 1 pmol/μL internal standard (corticosterone-d8 in acetonitrile) were added to each sample. Samples were afterwards thoroughly mixed and incubated for 22 h (220 rpm, 37 °C). Enzymatic activity was halted by adding 150 μL of 10% sodium carbonate (pH 9.6). Subsequently, liquid–liquid extraction was performed by adding 2 mL of methyl tert-butyl ether, followed by 10 min of mixing (orbital: 60 rpm, 45 s; reciprocal: 90°, 15 s; vibro/pause: 1°, 3 s). Sample centrifugation for 10 min (260× *g*, RT) followed. Samples were then stored at −20 °C for at least 30 min to induce a clear phase separation. A 1 mL aliquot of the ether phase was fully evaporated (no heating, pulse vent: 1; vacuum concentrator: Jouan RC 10.11, RCT 90, Gemini B.V., Apeldoorn, Netherlands; vacuum controller: PVK 610, Vacu-Box, MLT AG Labortechnik, Wangen, Switzerland). The residue was reconstituted in 500 μL of 80% acetonitrile/0.1% formic acid, sonicated for 5 min, and vortexed thoroughly. Finally, 100 μL were transferred to an HPLC vial for LC-MS/MS analysis.

### 4.5. Liquid Chromatography Tandem-Mass Spectrometry Analysis

The extracted corticosterone was quantified using an Agilent Infinity 1260 HPLC system (1260 Binary Pump, 1260 Infinity II Multisampler, 1260 Infinity II Multicolumn Thermostat, 1260 Infinity II Diode Array Detector, and an Agilent instrument control framework; Agilent Technologies, Inc., Waldbronn, Germany). The autosampler tray temperature was maintained at 4 °C. The HPLC was combined with a Z spray electrospray ionization interface tandem-mass spectrometry system. The mass spectrometer was equipped with an Agilent 6470 Quadrupole LC/MS (1260 II Infinity LC system; Agilent Technologies, Inc., Waldbronn, Germany). Nitrogen was used as the collision gas. Chromatographic data were processed using the Skyline software (https://skyline.ms/project/home/software/skyline/begin.view (accessed on 6 November 2024)), with each urine sample analyzed in duplicate by LC-MS/MS [45].

### 4.6. Quantification of Corticosterone

**Figure 3:**
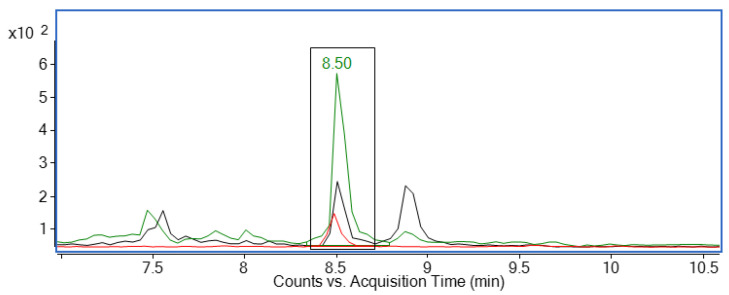
MRM chromatogram of corticosterone and corticosterone-d8. Green line: *m*/*z* 347.2 > 121.1 (qualifier of corticosterone), black line: *m*/*z* 347.2 > 97.1 (quantifier of corticosterone), red line: *m*/*z* 355.3 > 100.2 (quantifier corticosterone-d8).

Corticosterone in mouse urine was quantified based on the isotope-dilution approach. The quantifier mass transition of corticosterone (*m*/*z* 347.2 > 97.1) was utilized for quantification in relation to the quantifier mass transition of the deuterated internal standard (*m*/*z* 355.3 > 100.2, see Figure 3). Peak areas of corticosterone were normalized to the peak area of the deuterated analogue. For method validation, matrix-matched samples were prepared. Matrix-matched samples implied the extraction of urine pool samples according to the previously described extraction protocol (see Section 4.4) without the addition of corticosterone-d8 (10 µL of 100% acetonitrile instead). Corticosterone-d8 was added when resuspending the samples at the end of the sample preparation. The matrix effect was thus considered.

### 4.7. Quantification of Creatinine

Creatinine in mouse urine was determined with an enzymatic creatinine kit from Thermo Fisher Scientific Inc. (Indiko™ und Konelab™ System reagents for clinical chemistry, acid phosphatase to creatinine, Waltham, MA, USA). Urine samples were diluted 1:16 with HPLC water. We pipetted 200 µL of reagent A and 100 µL of reagent B into each reaction vessel. Then, 5 µL of the diluted urine was added. An incubation at 37 °C for 15 min followed (100 rpm). We transferred 80 µL of treated samples into each well of the microtiter plate in triplicate. The microtiter plate was measured at 37 °C and 540 nm.

### 4.8. Method Validation

#### 4.8.1. Linearity of Detection, LOD, LOQ

To evaluate linearity, corticosterone-d8 standard was prepared at concentrations of 1, 100, 500, 1000, and 5000 fmol/µL in matrix-matched samples in triplicate. Calibration curves were afterwards subjected to linear regression analysis.

The LOD and LOQ was calculated based on the quantifier of the internal standard (*m*/*z* 355.3 > 100.2). A calibration curve was prepared at concentrations of 1, 2, 10, 20, and 40 fmol/µL in matrix-matched samples in triplicate. The SNR was plotted against the applied analyte concentrations. The criterion for the LOD and LOQ implied an SNR exceeding 3 and 10, respectively. SNR was determined with the Agilent MassHunter Workstation Software Qualitative Analysis Version 10.0 (signal definition: height, noise definition: root-mean-square (RMS), specific noise regions: 8.0–8.2 min).

#### 4.8.2. Recovery, Intra- and Inter-Day Variation

The recovery was calculated by comparing the corticosterone-d8 peak area (10 µL of 1 pmol/µL reagent) in extracts of the same urine pool. The urine samples were either spiked with the internal standard before extraction or after extraction. Intra-day variation was determined by quantification of corticosterone in three separate extracts of urine pool samples that were prepared and analyzed on the same day in triplicate. This setup was repeated for three randomly selected days. For inter-day variation, urine pool samples were extracted at three different days in triplicate. The data collected for the intra-day variation were also utilized for calculations of the inter-day variation. The variation acceptance criterion for each urine pool sample was ≤15%.

#### 4.8.3. Accuracy and Matrix Effect

A solvent calibration curve (80% acetonitrile/0.1% formic acid) and a matrix calibration curve (mouse urine pool) was prepared. Both calibration curves were spiked with 1, 2.5, 5, 7.5, and 10 µL of the internal standard corticosterone-d8 (1 pmol/µL), but only the matrix (urine) samples were submitted to extraction (see Section 4.4).

The solvent calibration curve was utilized for accuracy and matrix effect calculations. The matrix influence was calculated using the slope of the linear regressions: $Matrix\;effect\left(\%\right)=\left(\frac{Slope\;of\;matrix-match\;edcurve}{Slope\;of\;solvent\;curve}\right)\times$ 100. The total ion chromatogram was used for this analysis except for *m*/*z* 355.3 > 337.5. Each experiment was performed in duplicate.

#### 4.8.4. Stability of Extracts

The stability of corticosterone in eight separately extracted samples of the same urine pool stored at −80 °C was determined. Long-term storage (28 d to 34 d) of extracts was checked as the mass spectrometers are often operating at full capacity.

### 4.9. Statistical Analyses

Plots were generated by GraphPad Prism (version 6; Graphstats Technologies Private Limited, Bangalore, India). Statistical analyses were performed using IBM^®^ SPSS^®^ Statistics (version 20; IBM Deutschland GmbH, Ehningen, Germany). Differences between two groups were studied by application of an independent-samples t test (normal distribution of data, two-tailed) or Mann–Whitney U test. Statistical significance between tested groups was accepted when * *p* ≤ 0.05, ** *p* ≤ 0.01, *** *p* ≤ 0.001, and **** *p* ≤ 0.0001, trends were defined when 0.05 < *p* < 0.1.

## 5. Conclusions

The developed and validated LC-MS/MS method for quantification of corticosterone in mouse urine enabled the measurement at femtomole per microliter level. Enzymatic hydrolysis of urine samples with β-glucuronidase from *E. coli* was included in the extraction protocol following a simple liquid–liquid extraction with methyl tert-butyl ether. This innovative method can be conducted with a high sensitivity as well as reproducibility for monitoring stress levels of mice. Our established method represents a suitable alternative to the collection of feces and subsequent analysis via enzyme immunoassays, which is currently more frequently applied. In order to ensure high scientific standards and therefore reliable outcomes, animal welfare should always be taken into account, as both variables are interdependent. We support the monitoring of stress levels for laboratory animals, that are actively used in experiments, including (1) a frequent and non-invasive sampling method, (2) a suitable sample matrix, (3) an adequate sample preparation, (4) and accurate quantification of the stress hormone of interest.

## Figures and Tables

**Table 1:** Tested amounts of β-glucuronidase for enzymatic hydrolysis of corticosterone glucuronide in urine.

Amount of β-Glucuronidase [U] ^a^	Concentration of Corticosterone [fmol/µL] ^b^	Variation [%] ^c^
25	78.11 ± 3.7	7.9
50	83.57 ± 3.5	1.5
100	82.23 ± 3.9	3.1
200	84.82 ± 5.5	-
400	77.39 ± 3.0	8.8

^a^ The required amount of β-glucuronidase for deconjugation of glucuronidated corticosterone was determined using matrix-matched samples. ^b^ The data represent means ± standard deviation of 4 analyses. The concentration of corticosterone (*m*/*z* 347.2 > 97.1) was calculated in reference to the internal standard corticosterone-d8 (*m*/*z* 355.3 > 100.2). ^c^ The percentage variation for each tested amount of β-glucuronidase refers to the urine pool treated with 200 U enzyme.

**Table 2:** Liquid chromatography and compound-specific MRM parameters.

Column	Kinetex C8 (2.6 µm, 100 Å, 150 × 4.60 mm; Phenomenex Ltd., Aschaffenburg, Germany)					
Column temperature	40 °C					
Flow rate	0.450 mL/min					
Solvents	A	Water + 0.1% formic acid				
	B	Acetonitrile + 0.1% formic acid				
Gradient	1 min	20% B				
	8–9 min	100% B				
	9.01–12 min	20% B				
Postrun	4 min	20% B				
Injection volume	5 µL					
Source parameters	Mode	Positive				
	Capillary	4500 V				
	Drying gas temperature	120 °C				
	Drying gas flow (N_2_)	11 L/min				
	Nebulizer	40 psi				
	Nozzle	0 V				
	Sheath gas temperature	400 °C				
	Sheath gas flow (N_2_)	12 L/min				
Multiple reaction monitoring	Compound		Retention time [min]	MRM transition [*m*/*z*]	Collision energy [eV]	Fragmentor voltage [V]
	Corticosterone	Qualifier	8.50	347.2 > 121.1	30	125
		Quantifier		347.2 > 97.1	40	160
	Corticosterone-d8	Qualifier	8.48	355.3 > 125.0	30	125
		Quantifier		355.3 > 100.2	40	160
	Dwell time	175 ms				
	Cell accelerator voltage	5 V				

**Table 3:** Method validation parameters determined for quantification of corticosterone in mouse urine samples.

Parameter ^a^	Corticosterone
Linearity in range 1–5000 fmol/µL [r^2^] ^b^	0.988
Limit of detection (LOD) [fmol/µL] ^c^	0.358
Limit of quantification (LOQ) [fmol/µL] ^d^	0.823
Intra-day variation day 1 [%] ^e^	5.23 ± 0.1
Intra-day recovery day 1 [%] ^e^	90.4 ± 3.2
Intra-day variation day 2 [%] ^e^	5.59 ± 0.1
Intra-day recovery day 2 [%] ^e^	110.6 ± 3.6
Intra-day variation day 3 [%] ^e^	4.21 ± 0.1
Intra-day recovery day 3 [%] ^e^	98.4 ± 2.8
Inter-day variation [%] ^e^	7.34 ± 0.1
Inter-day recovery [%] ^e^	99.8 ± 9.1

^a^ Method validation parameters were determined using matrix-matched samples. ^b^ Urine extracts were spiked with deuterated internal standard at five different concentrations. The peak areas of corticosterone-d8 were plotted against the concentrations of corticosterone-d8. A linear regression analysis was conducted. ^c^ Defined as deuterated internal standard amount that produces a peak with a SNR of 3. ^d^ Defined as deuterated internal standard amount that produces a peak with a SNR of 10. ^e^ Data represent means ± standard deviation of 3 analyses (intra-day variation) or nine analyses (inter-day variation).

## Data Availability

The data that support the findings of this study are available from the corresponding author upon reasonable request.
